# Patterns of diet and anemia during pregnancy in the Province of Sidi Kacem, Morocco

**DOI:** 10.11604/pamj.2024.48.152.42410

**Published:** 2024-08-05

**Authors:** Najoua El Aski, Fatima Ouasmani, Rahma Erahioui, Samir Bikri, Abdelhalim Mesfioui

**Affiliations:** 1 Laboratory of Biology and Health, Faculty of Science, Ibn Tofail University, Kenitra, Morocco; 2 Higher Institute of Nursing Professions and Health Techniques, Rabat, Morocco,; 3 Laboratory of Natural Resources and Sustainable Development, Faculty of Science, Ibn Tofail University, Kenitra, Morocco

**Keywords:** Dietary habits, anemia, pregnant women, Sidi Kacem, consumption

## Abstract

**Introduction:**

the objective of this study is to examine dietary habits and certain cooking practices, evaluating their influence on the prevalence of anemia in pregnant women and exploring other factors associated with anemia in Morocco, specifically in the province of Sidi Kacem.

**Methods:**

the research utilized a survey conducted over a one-year duration, spanning from August 01, 2018, to August 01, 2019, employing a cross-sectional, and analytical methodology. The prevalence of anemia in pregnant women was determined based on complete blood count tests, using hemoglobin (Hb) levels and World Health Organization (WHO) thresholds. An anonymous structured questionnaire was used to assess the dietary practices of the participants. The impact of these practices on anemia was analyzed using multivariate logistic regression models. Data analysis was conducted using Statistical Package for the Social Sciences (SPSS) software version 26.

**Results:**

the anemia rate among pregnant women in the province of Sidi Kacem was 43.03%. The findings of the binary logistic regression analysis revealed a statistical association between anemia, the main sources of iron in the diet, the frequency of consumption of caffeine-containing beverages (tea, coffee) with meals, the use of specific cooking techniques that could influence the iron content of foods, and the daily diet.

**Conclusion:**

the prevalence of nutritional challenges in Morocco poses a barrier to human development and enhanced health outcomes. A comprehensive understanding of the severity of these deficiencies is imperative for tailoring interventions that are contextually relevant to each region within the country.

## Introduction

Anemia constitutes a widespread public health concern, having significant impacts on both health and socio-economic development. Although its prevalence in urban, peri-urban, and especially rural areas lacks reliable data, it is likely that in resource-limited regions, a significant number of children and women of childbearing age suffer from anemia [1]. In Morocco, few national or regional surveys indicate that anemia remains a persistent health problem, especially among women. The prevalence of iron-deficiency anemia is 37.2% in pregnant women and 32.6% in women of childbearing age [2]. Given the global magnitude of this condition, many countries, including ours, undertake interventions such as fortifying flour with elemental iron to reduce it, particularly among the most vulnerable groups. To assess the effectiveness of these interventions and the relevance of the strategies implemented, gathering information on the prevalence of anemia and its associated factors in specific contexts is crucial. Indeed, the determinants of anemia vary depending on socio-economic, demographic, and cultural contexts, including factors such as the mother's age, gestational age, activity and education, closely spaced pregnancies, prenatal care, parity, gravidity [3], nutritional status at the beginning of pregnancy, regularity of iron and folic acid intake, and diet [4]. Anemia, as a significant risk factor, is associated with notable complications in maternal and fetal morbidity, including intrauterine growth retardation, prematurity, and perinatal mortality [5].

In Moroccan and African traditions, excessive tea consumption could also be considered a risk factor [6]. According to the Moroccan Ministry of Health, postpartum hemorrhage represents the main cause of maternal death in 30% of cases in hospitals, with anemia identified as a risk factor. Iron deficiency anemia is the most widespread form of anemia globally. It indicates anemia due to iron deficiency, whose etiology is a lack of dietary intake or poor absorption of iron. It can be corrected by supplementation and a specific iron-rich diet [1]. In the Moroccan context, and more specifically in the province of Sidi Kacem, the dietary habits of pregnant women play a crucial role in the prevalence of iron deficiency anemia. Traditionally, the Moroccan diet, rich in vegetables, fruits, and cereals, could be a potential source of iron. However, the combination of insufficient consumption of iron-rich foods and the low bioavailability of plant-based iron can contribute to inadequate iron intake. Moreover, certain cultural and economic aspects may influence dietary choices, thus limiting access to highly bioavailable iron sources like red meat. Therefore, promoting a diet specifically rich in iron among this population, accompanied by iron supplementation if necessary, could significantly contribute to reducing the prevalence of iron-deficiency anemia in pregnant women, underscoring the importance of targeted nutritional interventions in managing anemia during pregnancy. The main objective of this research is to estimate the prevalence of anemia among pregnant women in the province of Sidi Kacem, Morocco, and to determine the dietary practices of these women while analyzing the impact of these practices on the occurrence of anemia.

## Methods

**Study framework:** Sidi Kacem is one of the four provinces within the Rabat-Salé-Kenitra region, as per Morocco's regional administrative division. It is further subdivided into 24 rural communes and 5 urban communes [7]. The most recent census [8] reports a population of 533,788 for the province, with nearly 66% residing in rural areas. Regarding education, the province of Sidi Kacem reports an illiteracy rate of 40.4%. This rate rises to 50.8% in rural areas, contrasting with the 31.0% recorded in urban areas, and it highlights a total of 51.8% illiterate women [9]. Consequently, poverty and vulnerability rates in the province are among the highest nationwide, with values reaching 8.77% and 25.20%, respectively [10].

**Type of study:** the survey was conducted over a one-year period, spanning from August 01, 2018, to August 01, 2019, utilizing a cross-sectional, and analytical approach.

**Study population:** the study included all pregnant women (416) who attended antenatal appointments at the birthing centers and health centers selected for the study. These centers accounted for more than two-thirds of the pregnant women in the province attending these appointments during the study period; these centers were chosen by random draw. The antenatal appointments aimed to screen for and prevent maternal and fetal complications and to treat them in a timely manner.

**Sampling and sample size:** sampling was conducted consecutively, and the sample size was determined using the Lorenz formula [11]. The reported prevalence of anemia among pregnant women in Morocco, as indicated by the Ministry of Health in 2000 through the National Survey on Iron Deficiency Anemia, was 37% [12]. Utilizing this prevalence, the calculation yielded a minimum sample size of 358 patients.


$$n=t^{2}\times p\times \frac{\left(1-p\right)}{m^{2}}$$

Where n: minimum sample size required to achieve significant results for an event at a set risk level. T: confidence level (the standard value for a 95% confidence level is 1.96); p: estimated proportion of the population exhibiting the characteristic; m: margin of error (generally set at 5%); n= 3.84x 0.37x0.63/0.05x0.05= 358.

**Inclusion and exclusion criteria:** we enrolled all pregnant women attending prenatal consultations within the pregnancy and childbirth monitoring program or those presenting for childbirth who underwent a complete blood count (CBC). Pregnant women without a CBC were excluded from the study.

**Data collection and analysis:** the prevalence of anemia was determined based on complete blood count tests performed by the participants, focusing on hemoglobin (Hb) levels. The WHO thresholds for anemia in pregnant women were used to classify participants as anemic or non-anemic: women with an Hb level < 11 g/dl were considered anemic, and those with an Hb level > 11g/dl were considered non-anemic [13]. Considering the degree of severity, the distinctions made were as follows: mild anemia with an Hb level between 9.1 and 11g/dl, moderate anemia with a Hb level between 7 and 9 g/dl, and severe anemia with an Hb level below 7g/dl. A normal mean corpuscular volume (MCV) was defined within the range of 80 to 100 µ3. Microcytosis was identified below 80 µ3, while macrocytosis was observed above 100 µ3. Hypochromia was characterized by a mean corpuscular hemoglobin content (MCHC) < 27 pg, whereas normochromia was defined by an MCHC greater than or equal to 27 pg. The dietary practices of pregnant women were measured using an anonymous structured questionnaire administered during face-to-face interviews. The questionnaire included questions about the frequency of consumption of different food groups and the dietary habits of these women. The responses were coded and analyzed to identify dietary trends. The impact of dietary practices on the occurrence of anemia was analyzed using multivariate statistical models. Logistic regressions were used to evaluate the association between different dietary practices and the presence of anemia. Our data analysis was conducted using SPSS software version 26.

**Ethical concerns:** we obtained authorization from the medical delegate of the Ministry of Health and Social Protection in the province of Sidi Kacem, who granted approval for the study to proceed. Additionally, each participant provided informed consent for inclusion in the study. To maintain confidentiality, data collection forms were coded. Subsequently, the data underwent thorough and anonymous analysis.

## Results

**Socioeconomic and demographic profile of participants:** the distribution of pregnant women by age group revealed a notable predominance in the 25 to 35 age group, constituting 50% of the total. This age group was the most affected by anemia, with 64.24% of women experiencing anemia. Following this, the 18 to 25 age group had a 19% anemia rate, and the over 35 age group showed a rate of 16.8% of anemic women. Additionally, anemic women were more likely to originate from rural areas compared to urban areas, with a proportion of 62.6%. The average number of children was 1.25 (±1.37), ranging from a minimum of 0 to a maximum of 4. A significant portion, 62.02%, were illiterate, 35.81% had received primary or secondary school education, and a mere 2.16% had attended university. Eighty-eight point seventy (88.70%) were housewives with a low socioeconomic level. All the anemic women who took part in this study were married at the time of the survey. In addition, 33.89% of all women were primi. Regarding gravidity, it was observed that 33.89% of the women were in their first pregnancy, 43.27% were in their second pregnancy, and 22.84% were multigravida. Gestational age was categorized by weeks of amenorrhea, with 13% of women in the first trimester of pregnancy at the time of the survey, 43.7% in the second trimester, and 43.7% in the third trimester. Among women with fewer and multiple pregnancies, 56.1% had a shortened inter-pregnancy interval of 1 to 2 years. Breastfeeding was practiced by 74.1% of women. All surveyed women received regular monitoring in the province's health centers, and only 5% had anemia prior to pregnancy.

**Prevalence of anemia:** out of the 416 pregnant women included in our study sample, 179 were diagnosed with anemia, indicating a prevalence of 43.03% among pregnant women in Sidi Kacem from the beginning of August 01, 2018, and August 01, 2019. An analysis of the distribution based on the severity of anemia, following the WHO classification [14], revealed that the mild form was the most prevalent with 102 women or 56.98%, followed by the moderate form by 72 women or 40.22%, with the severe form being less frequent at only 5 women or 2.79%. However, it is crucial to emphasize the necessity for enhanced surveillance in this study population, where the prevalence of anemia is nearly half.

**Anemia type:** the conclusions drawn from the characterization of anemia, based on mean corpuscular hemoglobin (MCHT) and graft-versus-myeloma (GMV) levels, reveal closely distributed results between hypochromic and normochromic forms, accounting for 47.49% and 52.51%, respectively. Based on GMV, normocytic anemias were prevalent at 67.04%, followed by microcytic anemias at 31.28%, with macrocytic anemias being exceptionally rare at 1.68%. The combination of GMV and MCHT (Table 1) indicates the predominance of the Normochromic, Normocytic form at 50.84%, followed by the Hypochromic, Microcytic form at 31.28%, then the Hypochromic, Normocytic form at 16.20%, and finally the Normochromic, Macrocytic form at 1.67%. The amalgamation of both criteria, GMV and MCHT, uncovered a prevalence of normocytic normochromic anemias, followed by microcytic hypochromic anemias, normocytic hypochromic anemias, and finally, macrocytic normochromic anemias, which were exceedingly rare.

**Table 1:** anemia by combination of the two criteria hematopoietic stem cell transplantation and graft-versus-myeloma

Hypochromic, microcytic		Hypochromic, normocytic		Normochromic, normocytic		Normochromic, macrocytic	
Number	%	Number	%	Number	%	Number	%
56	31.28	29	16.20	91	50.84	3	1.67

Hypochromic, Microcytic: MCHT < 27 pg et GMV < 80 µ3; Hypochromic, Normocytic: MCHT < 27 pg et 80 µ3 ≤ GMV < 100 µ3; Normochromic, Normocytic: MCHT ≥ 27 pg et 80 µ3 ≤ GMV < 100 µ3; Normochromic, Macrocytic: MCHT ≥ 27 pg et GMV ≥ 100 µ3

**Factors related to anemia:** analysis of the dietary data for pregnant women with anemia revealed a notable consumption of cereal fats and a reduced intake of meat and dairy products. Specifically, all women included at least one type of cereal in their daily diet (such as Moroccan pancakes - Msemen, bread, wheat soup - Tchicha, or barley - Belboula, pasta, couscous, etc.) and one type of fat (table oil, olive oil). Furthermore, 73.18% adhered to a vegetarian diet primarily consisting of vegetables and legumes, while only 26.82% followed a balanced diet that included meat, fish, and vegetables simultaneously. Additionally, 49% of the women incorporated dairy products into their diet. In comparison with the non-anemic women group (Table 2), the Chi-square test indicated statistically highly significant differences in the primary sources of iron in the diet, the frequency of consuming caffeine-containing drinks (tea, coffee) with meals, the utilization of specific cooking techniques that might affect the iron content of food, and a significant difference in the daily diet. Moreover, none of the women were directed to a nutritionist for personalized dietary intervention. Nevertheless, in 72.84% of cases, women in our sample received nutritional guidance from health professionals, predominantly midwives, in the form of limited recommendations. This included encouragement to consume iron-rich foods like liver, spleen, lentils, green vegetables, and beets, as well as advice to refrain from drinking tea and coffee with meals. Notably, 42.3% of anemic women reported consuming 1 to 7 glasses of tea daily, with an average of 2.79 glasses per day.

**Table 2:** association between dietary habits and anemia

Variables	Modalities	Total effective	Women A		Women NA		P value
			n	%	n	%	
Your usual diet	Balanced (meat, fish, vegetables)	82	48	26.8	34	14.3	0.002*
	Mainly vegetarian	334	131	73.2	203	85.6	
How often do you eat iron-rich foods?	Several times per week	71	32	17.9	39	16.4	0.703
	Rarely	345	147	82.1	198	83.5	
The main iron sources in your diet	Pulses, green leafy vegetables, red meat	301	93	51.9	208	87.5	0.000*
	Poultry, Pulses	115	86	48.04	29	12,2	
Consumption of foods rich in vitamin C, which improves iron absorption	Yes	110	50	27.9	60	25,3	0.549
	No	306	129	72.06	177	74.7	
How often do you drink caffeinated beverages (tea, coffee) with meals?	Frequently	252	145	81	107	45.1	0.000*
	Occasionally	123	31	17.3	92	38.8	
	Rarely	41	3	1.7	38	16	
Use of specific cooking techniques that could influence the iron content of foods	Yes	376	179	100	197	83.1	0.000*
	No	40	0	0	40	16.9	

In the binary logistic regression model, the independent variables comprised the primary sources of iron in the diet, the frequency of consuming caffeine-containing beverages (tea, coffee) with meals, the utilization of specific cooking techniques that could impact the iron content of foods, and the daily diet (Table 3). The analysis indicates a positive association between anemia and these factors, suggesting that the primary sources of iron in women's diets, the frequency of consuming caffeine-containing beverages (tea, coffee) with meals, the utilization of specific cooking techniques that could impact the iron content of foods, as well as the daily diet, are all significant contributors to the prevalence of anemia. Therefore, anemia is more prevalent in these women, influenced by the primary sources of iron in their diet, the frequency at which they consume caffeine-containing beverages (tea, coffee) during meals, and their utilization of specific cooking techniques that could affect the food's iron content. Among these methods, excessive cooking at high temperatures and boiling are notable for causing significant loss of water-soluble nutrients such as folic acid, and vitamins B6 and B12, which are crucial for preventing anemia and ultimately impact the overall quality of their daily diet. However, when considering Wald's odds ratio (OR), the main sources of iron in the diet exhibited the highest score, followed by frequency of consuming caffeinated beverages (tea, coffee) during meals, and then the usual diet and finally, the consumption of foods rich in vitamin C improves the absorption of iron.

**Table 3:** binary logistic regression model variables and anemia

	B	Se	Wald	Ddl	P-value	Exp (B)
Your usual diet	1.549	0.315	24.250	1	0,000*	4.709
How often do you eat iron-rich foods?	0.374	0.486	0.594	1	0.441	1.454
The main sources of iron in your diet	-3.133	0.433	52.278	1	0.000*	0.044
Consumption of foods rich in vitamin C, which improves iron absorption:	-0.655	0.294	4.975	1	0.026*	0.519
How often do you drink caffeinated beverages (tea, coffee) with meals?	1.673	0.236	50.264	1	0.000*	5.326
Use of specific cooking techniques that may affect the iron content of foods	20.234	5859.859	0000	1	0.997	0.685
Constant	-21.190	5859.859	0.000	1	0.997	0.000

B: regression coefficient; SE: standard error; Wald: odds ratio; ddl: degree of freedom; *Level of significance at (p < 0.05)

## Discussion

Similar to many developing countries, Morocco grapples with elevated rates of iron-deficiency anemia [15]. The primary causes of iron deficiency in this population appear to be linked to a low dietary iron intake and its diminished bioavailability [16]. The prevalence of insufficient intakes and/or limited bioavailability of dietary iron is widespread in Morocco, primarily stemming from the prevailing monotonous diets dominated by plant-based foods with minimal inclusion of animal products and foods rich in vitamin C [17]. Our research highlights a significant anemia rate of 43.03%, indicating a substantial health concern in the province of Sidi Kacem. This prevalence surpasses rates observed in other regions of Morocco. Specifically, studies have reported prevalences of 14.4% in Casablanca [18], 16.8% in the city of Temara [19], and 30% in Marrakech [20]. This discrepancy can be partially elucidated by variations in living standards between urban regions, including large cities, and predominantly rural areas or provinces. The elevated anemia rate is attributed to the adverse effects of precarious living conditions on the population's health. Notably, the province of Sidi Kacem exhibits some of the highest poverty and vulnerability rates in the country, reaching 8.77% and 25.20%, respectively [21]. Furthermore, the labor supply in the province has decreased by six percentage points since 2004, moving from 50.7% to 44.7% in 2014. Additionally, the provincial unemployment rate saw an increase of 0.9 points during the intercensal period, rising from 13.9% in 2004 to 14.8% in 2014. These circumstances contribute to the emergence of nutritional problems and deficiency-related illnesses, underscoring the imperative to give special attention to cases of anemia in pregnant women in this province. These findings may also apply to the broader Moroccan population confronting precarious conditions and unfavorable socio-economic circumstances, particularly in rural areas.

Our findings align with those from other studies, reporting rates of 43.2% in Cameroon [22], 42.2% in Turkey, 45.4%, and 43.4% in Algeria [23]. However, these figures are notably higher than those documented in developed countries, where the prevalence of anemia during pregnancy typically remains below 20% [24]. Mild and moderate forms of anemia are the most frequently observed, consistent with findings in other regions of Morocco [1,25], Mauritania [26], and Algeria [27]. It's noteworthy that anemia during pregnancy, even in its mild stage, particularly when iron deficiency is the cause, poses a risk factor for preterm delivery and contributes to growth retardation in the newborn [28]. Hypochromic and microcytic anemias are prevalent in this sample, with macrocytosis being rare. Normocytic normochromic anemias and microcytic hypochromic anemias are predominant, indicating anemia due to iron deficiency, commonly referred to as iron deficiency anemia. This condition arises from insufficient dietary iron intake or poor iron absorption. Globally, iron deficiency anemia stands as the most prevalent form of anemia [29]. Addressing this condition can be achieved through iron supplementation and adopting a balanced diet rich in this essential element [30]. Considering this, Morocco has been executing an integrated program to address micronutrient deficiencies since 1986. This initiative encompasses the enrichment and fortification of widely consumed staple foods, micronutrient supplementation for vulnerable populations, nutrition education campaigns, and the reinforcement of nutrition-related health programs [31,32]. Despite these efforts, these strategies have not significantly mitigated the prevalence of micronutrient deficiencies uniformly across all regions. Challenges such as insufficient information on nutrition and healthy eating among the population, a shortage of specialized human resources in nutrition, ineffective coordination among various stakeholders, and limited engagement of associations dedicated to consumer health protection [33] hinder the success of these programs in Morocco.

Upon analyzing the diets of the women in our sample, we identified low consumption of foods rich in heme iron (dairy and animal products) but observed very high consumption of vegetables, cereals, and fats. In reality, the dietary pattern of these women reflects the traditional Moroccan cuisine centered around the consumption of family tajine, with or without meat, primarily composed of fats and vegetables and accompanied by bread [34]. This dietary model seems insufficient to meet the iron needs of women during pregnancy. Addressing this requires special attention to women's diets and regular dietary monitoring. Furthermore, 42.3% of the women in our study consumed tea or coffee, or both, more than four times a week, known for their inhibitory effect on non-haem iron absorption [35]. It is important to highlight that, despite the common use of certain culinary techniques such as hulling and soaking in their environment, pregnant women in our study were not aware of their significant role in enhancing the absorption of non-haem iron (by reducing phytates). Similar findings were also noted by Tessier *et al*. [36], who observed that very few Tunisian women of childbearing age were knowledgeable about the link between iron deficiency and anemia. This underscores the necessity for nutritional education, especially among women in general and pregnant women in particular, regarding the role of iron in the body and the benefits of maintaining a balanced, iron-rich diet. Research suggests that enhanced nutritional knowledge correlates with improved dietary habits and nutritional status [38].

Therefore, it is crucial to incorporate nutrition education into school curricula. Adolescent girls, who will likely become future mothers, need to acquire knowledge about selecting appropriate foods, combining them effectively, and employing suitable culinary techniques [38] to prevent or address iron deficiency. Numerous studies have highlighted the positive association between the consumption of animal products and fruits and improved health outcomes [38,39]. Animal products serve as excellent sources of zinc, vitamin A, and vitamin B12, contributing to enhanced bioavailability of iron and zinc. Similarly, fruits like oranges play a significant role in providing vitamin C, vitamin A, and folate. Traditional food preparation and cooking methods, such as boiling vegetables in water that is later discarded, result in substantial loss of soluble nutrients like folic acid, vitamins B6, B12, and C [40]. To minimize nutrient loss, it is advisable to prevent overcooking by adding vegetables to boiling water and covering pots, which helps reduce vitamin C oxidation. Additionally, extended cooking or deep-frying of animal products can diminish iron absorption. Conversely, soaking beans is a beneficial practice as it reduces their phytate content.

## Conclusion

Despite being a significant public health concern in Morocco, iron deficiency anemia has not received adequate attention in health policies. Understanding the dietary habits influencing the bioavailability of dietary iron and identifying risk factors for iron deficiency are crucial pieces of information. This knowledge can assist Moroccan authorities and non-governmental organizations (NGOs) in more effectively targeting primary prevention programs for iron deficiency.

### What is known about this topic

- *Iron deficiency is a common cause of anemia in pregnant women;*
- *Anemia in pregnant women remains a significant public health concern; prevalence rates vary depending on global regions and socio-economic conditions;*
- *In Morocco, limited data are available on anemia among pregnant women and its associated factors*.

### What this study adds

- *Propose culturally sensitive interventions and educational approaches to address anemia in diverse populations of pregnant women;*
- *Offer additional avenues for exploration and research in the broader context of dietary habits and anemia among pregnant women*.
